# Perioperative Management and Surgical Outcomes of Colorectal Cancer Patients Undergoing Peritoneal Dialysis for End-Stage Kidney Disease

**DOI:** 10.7759/cureus.26708

**Published:** 2022-07-09

**Authors:** Nobuji Kouno, Ryo Takahashi, Takumi Furuya, Takahisa Fujikawa

**Affiliations:** 1 Surgery, Kokura Memorial Hospital, Kitakyushu, JPN

**Keywords:** end-stage kidney disease, end-stage renal disease, surgical outcome, perioperative management, peritoneal dialysis, colorectal cancer surgery

## Abstract

Introduction: Despite the fact that the number of peritoneal dialysis (PD) patients is increasing, there is little evidence on the surgical outcomes of PD patients who have colorectal cancer surgery, and there is no consensus on the safety and practicality of continuing PD.

Methods: We retrospectively evaluated the short- and long-term results, as well as the feasibility of continuing PD, in eight patients with PD who had colorectal cancer surgery at our institution between January 2010 and January 2021.

Results: The scheduled open-fashioned resection was performed in one patient, whereas the other seven surgeries were all conducted laparoscopically, with no intraoperative conversion to laparotomy necessary. Except for one patient with a history of recurring PD-related peritonitis, the PD catheter was kept in seven of the eight cases. Five of the seven patients continuing PD underwent temporary postoperative hemodialysis. At a median of 24.5 months of postoperative monitoring, no infectious complications were observed, six cases continued PD, and no recurrence of colorectal cancer was observed in all cases.

Conclusions: Routine curative-intent colorectal cancer surgery with the preservation of the PD catheter is possible and safe in individuals receiving PD. This patient population's short- and long-term oncological results are comparable to general surgical outcomes of those without chronic kidney disease. PD can be maintained for a long period of time following major colorectal cancer surgery.

## Introduction

Hemodialysis (HD) is used by the majority of chronic dialysis patients in Japan, with less than 3% using peritoneal dialysis (PD), which is lower than the global prevalence of 11%. In Japan, however, PD accounted for 5.6% (2,293/40,468) of all new chronic dialysis therapy introductions in 2018, and the number of PD patients has risen since 2017 [1]. Because of its limited influence on circulatory dynamics [2] and efficiency in maintaining residual renal function [3], PD is useful and expected to become more widely used.

Malignant tumors account for about 9% of fatalities in chronic HD patients [1], and gastroenterological malignancies such as colorectal cancer can also occur in PD patients. However, there is little information on these patients' perioperative treatment and surgical outcomes, particularly about the timing of postoperative transition and resumption of PD. Our institution, a tertiary referral hospital, has been actively offering PD to new dialysis patients, despite the fact that the PD introduction rate in Japan varies substantially [1]. As a result, we experienced a relatively high frequency of colon cancer surgeries among PD patients.

The goal of the present study is to investigate the short- and long-term surgical outcomes of individuals who have received PD, as well as the feasibility of continuing PD following colon cancer surgery.

## Materials and methods

We searched the prospectively collected surgery database of a single institution for relevant cases from January 2010 to January 2021. Eight consecutive PD-conducted patients with end-stage kidney disease who underwent colorectal cancer surgery were included in this study, and patients who underwent emergency surgery were excluded. Electronic medical records of the patients from our institution were reviewed.

Preoperative lower gastrointestinal endoscopy and biopsy revealed all patients to have colorectal cancer, which was treated with curative-intent surgery. The severity of patient symptoms and level of patient functioning in terms of ambulation were reported according to the Eastern Cooperative Oncology Group scale of performance status [4]. Postoperative complications were assessed and categorized according to the Clavien-Dindo (CD) classification, and CD class 2 or higher was considered significant [5]. Operative mortality was defined as death within 30 days after surgery. Complete resumption of PD was defined as the return to the same frequency and volume of infusion, diffusion, and drainage as before surgery.

The clinical background, short- and long-term surgical results, PD catheter preservation and removal during surgery, temporary HD transition, and the time of PD restart were all retrospectively examined.

## Results

Clinical background of the included patients

The clinical background data of the eight included patients are shown in Table 1. In PD patients undergoing colorectal cancer surgery, there were five males and three females. The median age at surgery was 74 years (range: 57-80 years), the performance status was 0 in two patients and 1 in six patients, and the median body mass index was 22.8 (range: 21.6-31.8). Four cases had diabetic nephropathy, two cases had hypertensive nephrosclerosis, and one case had IgA nephropathy, which led to the initiation of dialysis. Five of the patients had atherosclerotic illnesses, such as cerebral infarction, internal carotid artery stenosis, angina pectoris, and myocardial infarction, and two of the patients had malignant diseases of other organs (lung cancer and hepatocellular carcinoma). Aside from the PD catheter insertion, three of the patients had previously undergone appendectomy, and one had previously undergone open cholecystectomy (Table 1).

**Table 1:** Clinical background of the patients in the current study. PD, peritoneal dialysis; PS, performance status; ESKD, end-stage kidney disease; PDP, peritoneal dialysis-related peritonitis; DN, diabetic nephropathy; IgAN, IgA nephropathy; HTNS, hypertensive nephrosclerosis; CI, cerebral infarction; MI, myocardial infarction; Af, atrial fibrillation; HCC, hepatocellular carcinoma; LC, lung cancer; ICS, internal carotid artery stenosis.

Case	Age	BMI	Gender	PS	ESKD course	PD duration (months)	Comorbidities	History of PDP	History of laparotomies	Clinical stage
1	80	23.2	M	1	DN	9	CI, MI, Af	No	None	Stage I
2	71	24.1	M	1	IgAN	3	None	No	None	Stage 0
3	77	20.8	F	1	DN	35	None	Yes	Appendectomy	Stage I, Stage 0
4	73	21.7	M	1	Uncertain	59	MI, LC	Yes	None	Stage I
5	78	27.3	M	0	HTNS	2	ICS	Yes	Appendectomy	Stage I
6	57	21.6	F	1	DN	3	HCC	No	Appendectomy	Stage I
7	74	31.8	F	1	HTNS	7	ICS	No	None	Stage I
8	74	22.4	M	0	DN	24	MI	Yes	Open cholecystectomy	Stage IVa

Before colorectal cancer surgery, the median duration of PD was nine months (range: 3-59 months). A history of PD-related peritonitis was seen in four patients. The preoperative diagnosis of colorectal cancer included Stage 0 in one individual, Stage I in five, two lesions (Stage 0 and I) in one, and Stage IVa (advanced sigmoid colon cancer with a single resectable lung metastasis) in one case (Table 1). Most patients in the current study were diagnosed at early stages, mainly because most of them received close medical follow-up for the underlying atherosclerotic diseases such as cardiovascular or cerebrovascular diseases.

Surgical results and short-term outcomes

**Figure 1:**
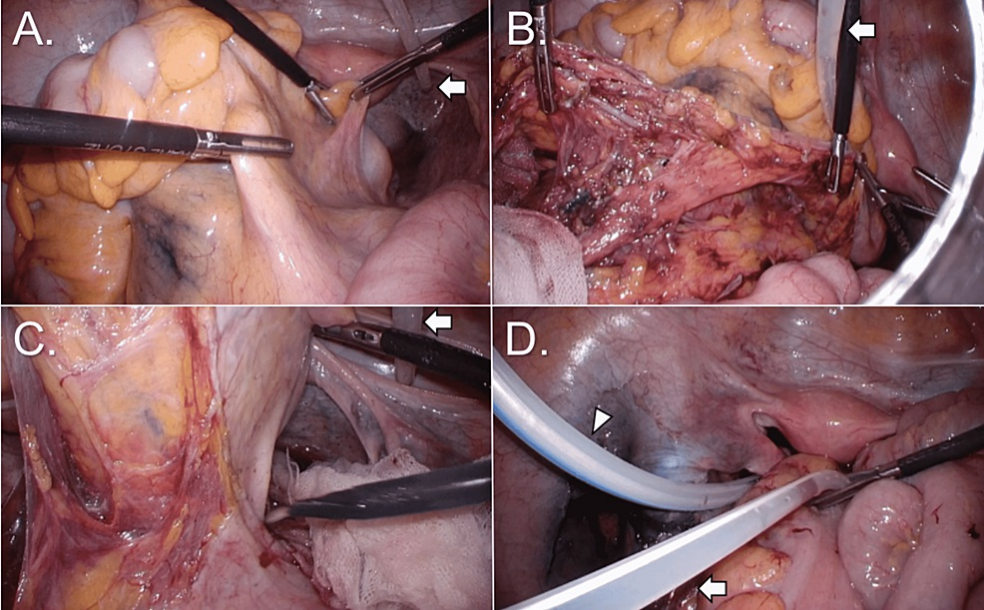
Intraoperative findings from performing a laparoscopic left hemicolectomy of the colon (case 6). Surgical procedures were done without much difficulty. (A) Preparing for medial dissection of sigmoid colon and rectum. (B) Status after medial dissection and lymph node dissection. (C) Dissecting mesorectum. (D) Relocating the PD catheter after putting an intraoperative drain in the pelvic cavity. Arrows indicate the PD catheter, and the arrowhead indicates an intraoperative drain.

In one case, an open sigmoid colon resection was performed as planned. The remaining seven surgeries were all done laparoscopically, and none of them required a laparotomy conversion during the procedure (Table 2). Low anterior resection without diverting ileostomy was performed in two cases, right hemicolectomy was performed in two cases, left hemicolectomy was performed in one case, left hemicolectomy + partial resection of the transverse colon was done in one case, and ileocecal resection was performed in one case. Appropriate lymph node dissection was performed according to the stage, and all patients obtained local R0 status. Intraoperative tests indicated no adhesions or changes in peritoneal morphology that would make surgery difficult due to PD in any of the patients. Only one patient with a frequent history of PD-related peritonitis had the PD catheter removed as planned. It seemed to be necessary to remove the catheter and re-insert the new one, but the patient was unwilling to do it. We decided to remove the catheter and transfer the patient to HD postoperatively. The other seven individuals were able to keep the catheter (Table 2). Figure 1 depicts the intraoperative findings of case 6, who underwent a laparoscopic left hemicolectomy.

**Table 2:** Short-term outcomes of peritoneal dialysis patients after colorectal cancer surgery. Lap, laparoscopy; ND, nodal dissection; CD, Clavien-Dindo classification; PD, peritoneal dialysis; POD, postoperative day; RHC, right hemicolectomy; LHC, left hemicolectomy; PTC, partial transverse colectomy; LAR, low anterior resection; ICR, ileocecal resection.

Case	Open or Lap	Procedure	ND	Operative time (minutes)	Blood loss (mL)	Complications (CD ≥ 2)	PD catheter	Pathological stage	Length of postoperative day (days)
1	Open	Sigmoidectomy	D2	155	60	-	Spared	Stage I	19
2	Lap	RHC	D2	165	3	-	Spared	Stage 0	16
3	Lap	LHC + PTC	D2	334	10	Anastomotic bleeding	Spared	Stage I, Stage 0	35
4	Lap	LAR	D2	300	60	Anastomotic leakage (minor)	Removed	Stage I	39
5	Lap	LAR	D2	289	40	-	Spared	Stage I	19
6	Lap	LHC	D2	267	35	-	Spared	Stage I	13
7	Lap	RHC	D3	252	29	-	Spared	Stage I	24
8	Lap	ICR	D3	309	54	-	Spared	Stage IVa (PUL)	16

The average operational duration was 278 minutes (range: 155-334 minutes), with a median blood loss of 38 ml (range: 3-60 ml). In either case, there was no serious intraoperative bleeding that necessitated a blood transfusion. Two patients, one with anastomotic leakage that could be managed conservatively and the other with anastomotic hemorrhage that needed endoscopic hemostasis, experienced postoperative complications of CD class 2 or higher (Table 2). The median length of stay in the hospital after surgery was 19 days (range: 13-39 days), which appeared to be longer than typical because it takes a certain number of days to properly restart PD following surgery.

Perioperative dialysis-related results in patients with continuous PD

Except for case 5, all seven subjects continued to have PD until the day before surgery. This is because we have not had a protocol with preoperative HD for PD patients; therefore, the preoperative management was sometimes varied. The patient with a frequent history of PD-related peritonitis (case 4) got the PD catheter removed intraoperatively and transferred to HD as planned postoperatively. With or without a daily catheter flush, all patients were able to continue PD. Although two patients were allowed to continue PD without postoperative HD because they had residual renal function and their urine volume was preserved to some extent, temporary HD was conducted in five cases before resuming postoperative PD (Table 3).

**Table 3:** Perioperative dialysis-related results in peritoneal dialysis patients. HD, hemodialysis; PD, peritoneal dialysis; POD, postoperative day.

Daily catheter flush (POD)	Postoperative HD	Date of drain removal (POD)	PD fluid leakage	PD complete resumption date (POD)	Preventive antibiotics use (POD)
Not done	Yes	6	No	14	0-4
1-6	No	No drain	No	13	0-2
10-12	Yes	6	Yes	21	0
-	Yes	No drain	-	-	0-14
10-13	Yes	5	No	14	0
1-6	No	4	No	7	0
Not done	Yes	5	No	14	0
Not done	Yes	No drain	No	8	0

Five patients had intraoperative drains, which were all removed by the sixth postoperative day. Although all seven subjects were able to resume PD after surgery, full resumption occurred between the seventh and 21st postoperative days (Table 3).

Preventive antibiotics (first-generation cephalosporin) for wound infection or catheter infection were used only on the operative day in five cases, were continued until postoperative day two in one case, and were continued until postoperative day four in one case. We did not check the peritoneum fluid culture in all cases. Antibiotics were used for 14 days in the case of anastomotic leakage.

**Figure 2:**
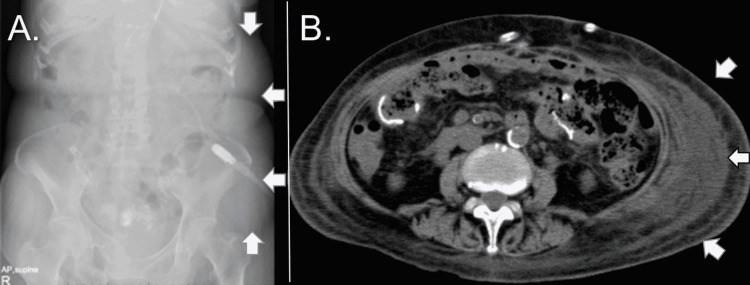
X-ray and CT images of subcutaneous edema after postoperative peritoneal dialysis resumption (case 3). (A) An abdominal X-ray shows the obviously low radiolucency area on the left abdomen (arrows). (B) The CT image shows subcutaneous edema spreading widely on the left lateral abdomen.

We had one case where a subcutaneous dialysate leak occurred after PD was resumed (case 3, Figure 2). For wounds of 12-mm trocars or small laparotomy wounds, wound closure was done in three layers (peritoneum + fascia + subcutaneous tissue), and for wounds of 5-mm trocars, wound closure was done in two layers (fascia-peritoneum + subcutaneous), and the intraoperative drain was placed but removed on the sixth postoperative day. On the 11th postoperative day, HD was completed, and PD was started; however, dialysis fluid drainage was inadequate. Abdominal X-ray and CT imaging revealed left-sided abdominal edema surrounding the drain removal site (Figure 2), which was treated conservatively.

Long-term results of the patients in the present cohort

In case 8, a colorectal cancer pulmonary metastasis was discovered preoperatively, but it was resected two months after the initial tumor was removed. During a median monitoring time of 31 months (range: 10-70 months), no recurrence of colorectal cancer was found in any of the patients (Table 4). Respiratory failure owing to primary lung cancer, cardiac arrest due to aortic stenosis, and hypoglycemia due to multiple organ failure were the reasons for death in three cases, all of which were caused by other diseases. Due to aggravation of heart failure and inadequate fluid management, PD could not be sustained over the follow-up period, and HD was introduced in one instance (case 1). PD has been continued in the remaining six instances, with a median observation length of 24.5 months (range: 10-46 months) (Table 4).

**Table 4:** Long-term outcomes of peritoneal dialysis patients after colorectal cancer surgery. CRC, colorectal cancer; PD, peritoneal dialysis; LC, lung cancer; AS, aortic valve stenosis; MOF, multiple organ failure; CHF, congestive heart failure; PDP, peritoneal dialysis-related peritonitis.

Case	Pathological stage	Observation period (months)	Recurrence of CRC	Prognosis (cause of death)	Complications related to PD	PD continuation
1	Stage I	58	No	Death (LC)	No	No
2	Stage 0	46	No	Death (AS)	No	Yes
3	Stage I, Stage 0	37	No	Death (MOF)	No	Yes
4	Stage I	70	No	Alive	No	No
5	Stage I	24	No	Alive	No	Yes
6	Stage I	25	No	Alive	No	Yes
7	Stage I	17	No	Alive	CHF, PDP	Yes
8	Stage IVa (PUL)	10	No	Alive	No	Yes

## Discussion

In the current study, we retrospectively evaluated the surgical outcomes and the feasibility of continuing PD in eight PD patients undergoing colorectal cancer surgery. All patients can have routine curative-intent cancer resections, and the PD catheter can be kept in most situations. PD was restarted on postoperative days eight to 21 in all seven instances with preserved PD catheters. At a median postoperative time of 24.5 months, no infectious problems were seen, PD could be continued in six cases, and no colorectal cancer recurrence was observed in all cases.

The global PD rate is around 11% [6]; however, it varies significantly from country to country [7]. In Japan, the number of chronic dialysis patients is rising year by year, surpassing 330,000, and the number of newly introduced patients is also increasing [1]; however, the vast majority of them are on HD, with just approximately 3% on PD. This is attributable to a significant number of HD-capable hospitals [8] as well as a scarcity of PD-experienced physicians and nurses in Japan [9]. There are many publications on surgery for urgent abdominal diseases in PD patients, such as studies on cholecystitis or appendicitis, but just a few papers on PD patients who had colorectal cancer surgery have been published thus far. These emergent surgeries and colorectal cancer surgeries are both contaminated surgeries. We consider that perioperative PD catheter management is basically similar.

Peritoneal degeneration and encapsulating peritoneal sclerosis are linked to the length of PD and a history of PD-related peritonitis [10]. Furthermore, due to excess fluid and protein loss in the dialysate, patients with PD are prone to malnutrition, which impairs wound healing [10], and bowel resection in patients with chronic renal failure has been linked to an increased risk of mortality [11]. Laparoscopic colorectal cancer surgery was shown to be feasible and safe in the majority of instances in the present investigation. The most significant reason for this preferable outcome is the tight connection between the department of nephrology and surgery in our institution. We keep in touch frequently concerning perioperative management, such as the timing of restarting PD, trouble management related to PD, and catheter management. In addition, we often assist the PD catheter insertion or relocation laparoscopically. These experiences enable us to have a good image of the surgical field where the catheter is located. Two clinically relevant postoperative complications occurred, including an anastomotic leak and bleeding, but the patients were otherwise well-managed under strict perioperative supervision.

There is no consensus on how to handle dialysis-related concerns during surgery, such as when to switch to HD or restart PD, or whether to keep PD catheters. Attard et al. proposed the following perioperative management of PD patients during right hemicolectomy [12]: (1) temporary HD transition before surgery, (2) preservation of the PD catheter, (3) administration of broad-spectrum antibiotics within 48 hours after surgery, (4) lavage of the PD catheter with fresh heparin every day from the first postoperative day until the resumption of PD, (5) frequent confirmation of blood tests until the resumption of PD, and (6) resumption of PD two weeks after surgery.

In terms of perioperative PD catheter preservation, Kosmadakis et al. reported that PD catheters were removed after gastrointestinal surgery for gastric cancer and colorectal cancer because of the risk of postoperative intra-abdominal infection [13]. In most instances, PD was prolonged until the day before surgery in the current research, and we feel that a preoperative HD transition is not always required. Furthermore, with the exception of one instance when the catheter was withdrawn as intended, we kept the catheter in the vast majority of patients, and there was no postoperative catheter infection or PD-related peritonitis. Although some studies suggest that intraoperative drain placement should be avoided as much as possible due to the risk of interfering with the PD catheter and causing dialysate leakage from the wound [14], informational drains may be used in many cases based on intraoperative findings during colorectal cancer surgery. The choice to keep the PD catheter should be based solely on the level of intraoperative contamination and adhesion, and we feel that it can be kept regardless of intraoperative drain placement because four of five patients with intraoperative drain could continue PD without infectious problems during monitoring periods in this study.

When it comes to the time of postoperative PD restart, Goel et al. found that PD-related peritonitis is frequently seen when PD is resumed soon after gastrointestinal surgery [15]. Furthermore, in patients with chronic renal failure, including PD patients, wound healing is delayed, and an increase in intra-abdominal pressure may interfere with wound healing when PD is restarted early after surgery [11,16]. Although the exact timing of resuming PD postoperatively is debatable, we feel that temporary HD at two to three weeks is suitable.

Periodic flushing with a small amount of new heparin [17] or intraperitoneal flushing with 500-1000 ml of dialysis solution [18] has been reported for postoperative PD catheter maintenance to prevent blockage. In the current study, three of the seven individuals that were able to continue PD without flushing or rinsing had no blockage. Although the distinction between flushing and rinsing in terms of avoiding blockage is unclear, it is preferable to minimize intra-abdominal pressure rise.

To the best of our knowledge, no information on the long-term results of colorectal cancer surgery in PD patients has been published. In the current study, there was no evidence of colorectal cancer recurrence in any of the patients, and the reason for death in three of the patients was other concomitant conditions rather than colorectal cancer. The curative effectiveness of surgery has been maintained, and in most cases, PD has been able to be prolonged over the long-term follow-up period. In general, intra-abdominal organ adhesion is to be predicted following abdominal surgery; however, postoperative PD can be safely prolonged for a long period after colorectal cancer surgery.

There are certain limitations to the current study. To begin with, the study was retrospective in nature; therefore, it is of limited use in determining the influence of treatments on outcomes. Second, various factors other than those described in this study might have contributed to colorectal cancer resolution, but they were outside the scope of our investigation. Third, the sample size was modest, and a greater number of instances would likely be beneficial in making more solid recommendations. Finally, a control group is necessary to demonstrate that our recommended management is preferable to alternative possibilities. To get deeper insights and generate substantial evidence, further large-scale prospective or retrospective investigations are required.

## Conclusions

In patients receiving PD, routine curative-intent colorectal cancer surgery with the preservation of the PD catheter is feasible and safe. This patient population's short- and long-term oncological results are comparable to general surgical outcomes of those without chronic kidney disease. Following major colorectal cancer surgery, PD can be maintained for a long time.
